# Social Media and the Empowering of Opponents of Medical Technologies: The Case of Anti-Vaccinationism

**DOI:** 10.2196/jmir.2409

**Published:** 2013-05-28

**Authors:** Kumanan Wilson, Jennifer Keelan

**Affiliations:** ^1^Ottawa Hospital Research Institute, University of OttawaDepartment of MedicineOttawa, ONCanada; ^2^University of TorontoSchool of Public HealthToronto, ONCanada

**Keywords:** social media, immunization

## Abstract

Social media has contributed positively to the interaction between proponents of medical products and technologies and the public by permitting more direct interaction between these two groups. However, it has also provided opponents of these products a new mechanism to organize opposition. Using the example of anti-vaccinationism, we provide recommendations for how proponents of medical products and technologies should address this new challenge.

## Introduction

Social media has been defined as “a group of Internet-based applications that…allow the creation and exchange of user generated content” [1]. These platforms range from social networking sites such as Facebook, to content sharing sites such as YouTube and Picasa, and even to interactive virtual worlds such as Second Life and World of Warcraft. Rapidly increasing in popularity and influence, social media presents a double-edged sword for proponents of medical technologies. On one hand, social media has transformed how companies communicate with potential consumers of medical pharmaceuticals and technologies. Both consumers and producers have a range of novel communication channels available to them that can rapidly match consumer interests and needs with available products and services. Social media platforms provide companies with new communication channels, relatively inexpensive and targeted advertising opportunities, and a consumer-mediated information stream that could potentially improve consumers’ trust in information and brand loyalty to companies through information shared online. Social media has also given consumers communications tools that enable them to rapidly seek health information, share medical advice, directly manage health conditions, and benefit from, and contribute to, a community discourse by rating, ranking, and describing experiences with medical products. These applications have been developed partly in response to a shift in how consumers see their role in managing their health in an increasingly complex and patient-oriented medical system [2-4].

However, along with these opportunities for empowering both health consumers and producers alike comes potential peril [5]. Social media activities have raised alarms in the medical research community over companies having more effective tools to directly market health products to consumers—an activity regulated in most jurisdictions outside the United States [6,7]. The direct marketing of pharmaceuticals, procedures, devices, and medical tests to consumers is thought to lead to overconsumption or inappropriate consumption of medical technologies [8,9]. Conversely, social media also presents new opportunities for opposition to medical technologies, most notably for those that raise the ire or concern of some citizens, such as religious opposition to stem cell or novel fertility technologies. Social media provides a new platform for these individuals to organize, communicate, and undermine industry messages. It allows these individuals to circumvent traditional communication mechanisms and therefore does not require their messages to be either acceptable or relevant to mainstream broadcasters. It thus permits a minority of motivated individuals to potentially control the discourse and, at times, contribute to the spread of misinformation, damaging an otherwise useful interaction between proponents and consumers. An example of where this disruptive new media has been particularly problematic, and which offers cautionary messages to advocates of other technologies, is in the field of immunization. We have been studying this phenomenon and provide a summary of our experiences and lessons for advocates of new and existing technologies.

## Social Media and the Anti-Vaccination Movement

Anti-vaccinationism has existed since the introduction of the first vaccine. Individuals who have alternate belief systems have mobilized, typically geographically, to communicate their concerns. This has led to sporadic vaccine rejection movements. More recently, the claim that the MMR (measles-mumps-rubella) vaccine or thimerosal containing vaccines are associated with autism continues to persist despite numerous studies refuting the link [10]. This rumor, largely initiated by a since-withdrawn paper in the *Lancet*, has resulted in vaccine rejection and contributed to over 26,000 cases of measles in Europe in 2011 [11,12].

**Figure 1:**
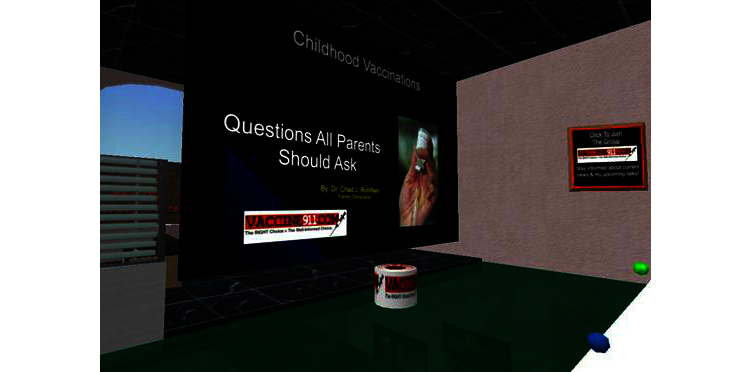
Screen capture from the Second Life Site of the Vaccine 911 auditorium: The Iowa Wellness and Spinal Tuning Center (SLurl 163,122,28; Image taken Dec 15, 2008).

What is social media’s role in all of this? Traditionally, geographic proximity was necessary for mobilizing anti-vaccination forces. However, social media has circumvented this potential barrier, allowing individuals from disparate regions who likely would not have otherwise communicated to come into contact. In this process, individuals who had otherwise had their viewpoints rejected and been marginalized can be emboldened and can feel empowered. Social media also provides these individuals with new dynamic mechanisms to communicate their viewpoints. We observed this in several ways while studying vaccine concerns. We first observed the congregation of anti-vaccination viewpoints on YouTube [13]. Individuals utilized YouTube to upload videos that highlighted vaccine concerns and commented on each other’s videos in a quasi social-network manner. Our observation was reinforced by the fact that anti-vaccination videos had more views and higher ratings than pro-vaccine videos. We observed similar vaccine concerns on the social media site MySpace when studying postings related to the HPV vaccine [14]. Examining these blogs revealed geographical clustering of anxiety—with Texas’ attempt to make the HPV vaccine mandatory leading to a plethora of anti-HPV vaccine blogs in that state. Our assessment of the blogging sentiments also revealed potential future challenges in having boys accept the vaccine given that boys’ blogs were determined to be more negative. We even observed organized anti-vaccine behavior when we surveyed health communications in the virtual world Second Life [15] (see Figure 1 for a screen capture showing the Vaccine 911 auditorium; Vaccine 911 is a vaccine critical organization that presents weekly lectures on immunization in Second Life).

As can be surmised, these pernicious activities can pose a real threat to mainstream messaging. If vaccination, one of the most important mechanisms for reducing mortality and morbidity where all established sources of information support the practice, can be undermined by social media activities, more novel technologies are at real risk of being similarly undermined [16]. Other examples of where traditional health messages have been undermined using social media include the promotion of anorexia and the spread of misinformation pertaining to rheumatoid arthritis [17,18].

## Recommendations

What strategies should advocates of medical technologies employ to combat the social mobilization of opposition to their products derived from marginalized opinions, hearsay, and inaccurate representation of the science involved? Here are a few recommendations stemming from our observations.

### Social Media Monitoring

This is now an established part of marketing strategies. Numerous services are available to track online comments and social media activity about a new product and also to analyze sentiment, providing businesses with an opportunity to interact with customers, and to potentially intervene and prevent viral marketing campaigns by responding rapidly to customer concerns [19]. This is a necessary first step for any pre-emption efforts. Moving beyond social media monitoring, advocates of medical technologies may also want to consider monitoring search behavior related to their products. In health, search term surveillance has shown promise in identifying behavior patterns and anticipating disease outbreaks [20].

### Be Where the Conversation Is

The US Centers for Disease Control and Prevention has made it part of their outreach mandate to master the various social media platforms so that, as much as possible, they can deliver scientifically accurate and appropriate content at the point when a consumer is seeking information, either via a Google keyword search, blogging on Facebook, watching videos on YouTube, or scanning related news items [21].

### Interacting Through Social Media

This is a delicate task that needs to be approached with caution. When exploring social media contact, proponents of medical products may encounter a lot of negative sentiment. However, responding to the sentiments may simply provide a platform and greater audience for the more extreme viewpoints. Proponents of medical products need to recognize that opposition to their products will lie along a spectrum. There will be those who are ideologically opposed, and no effort to persuade them will be successful and will likely only intensify their opposition. We observed this when studying anti-vaccination attitudes and found that individuals often frequent social media sites to hear like-minded viewpoints and are not interested in hearing alternate viewpoints [13]. While vaccination may be a somewhat extreme example given the intensity of rhetoric that characterizes the discussions, nanotechnology, stem cells, and reproductive technologies could also create similar opposition. Proponents of medical technologies need to recognize that their target is the ambivalent individual. An individual who has no strongly held opinion and is susceptible to influence by a persuasive argument or an argument that resonates with a strong pre-existing belief system they hold (for example religious/political views). This leads to our fourth recommendation.

### Recognizing the Power of Social Media

While some of the more radical viewpoints on social media may seem bizarre, dismissing the overall sentiments on social media would be a mistake. Public figures may champion these viewpoints (Jenny McCarthy on vaccines and Prince Charles on nanotechnology) giving the viewpoints’ credibility among more moderate participants. Further, heavy-handed tactics by proponents of new technology may backfire because of the ability to create opposition through social media. Finally, and perhaps most importantly, there may be a basis of truth to the concerns voiced on social media sites. Proponents of medical products would be well served to listen to this discourse, ignore the extreme contributors, and prepare to address the concerns of the more moderate contributors. Companies that respectfully acknowledge these concerns and respond with clear actions, demonstrating that these concerns are being listened to, will build trust in their products. In contrast, companies ignoring the media and its messages will do so at their own peril.

Social media has been described as a game changer and proponents of medical products will have to develop mechanisms to understand and manage its influence. In many ways, social media has been beneficial, serving to improve the interaction between proponents of products and the public, in addition to providing members of the public an opportunity to provide valid criticism. However, the risk of discourse being hijacked by an extreme minority can be destructive to the relationship between producer and consumer. Ours are but a few of the suggestions to guide proponents of medical technologies as they navigate this new media and its impact.
